# Pivotal Trial of the Neuroform Atlas Stent for Treatment of Anterior Circulation Aneurysms: One-Year Outcomes

**DOI:** 10.1161/STROKEAHA.119.028418

**Published:** 2020-06-17

**Authors:** Osama O. Zaidat, Ricardo A. Hanel, Eric A. Sauvageau, Amin Aghaebrahim, Eugene Lin, Ashutosh P. Jadhav, Tudor G. Jovin, Ahmad Khaldi, Rishi G. Gupta, Andrew Johnson, Donald Frei, David Loy, Adel Malek, Gabor Toth, Adnan Siddiqui, John Reavey-Cantwell, Ajith Thomas, Steven W. Hetts, Brian T. Jankowitz

**Affiliations:** 1 Neuroscience Department, Bon Secours Mercy Health St. Vincent Medical Center, Toledo, OH (O.O.Z., E.L.).; 2 Lyerly Neurosurgery, Jacksonville, FL (R.A.H., E.A.S., A.A.).; 3 The Stroke Institute, Department of Neurology, University of Pittsburgh Medical Center, PA (A.P.J.).; 4 Cooper University Hospital Neurological Institute, Camden, NJ (T.G.J.).; 5 WellStar Medical Group, Neurosurgery WellStar Health System, Marietta, GA (A.K., R.G.G.).; 6 Swedish Covenant Hospital Neurosurgery, Chicago, IL (A.J.).; 7 Radiology Imaging Associates, Swedish Medical Center, Englewood, CO (D.F.).; 8 Department of Radiology and Medical Imaging, University of Virginia, Charlottesville (D.L.).; 9 Department of Neurosurgery, Tufts Medical Center, Boston, MA (A.M.).; 10 Cerebrovascular Center, Cleveland Clinic, OH (G.T.).; 11 SUNY University at Buffalo, NY (A.S.).; 12 Virginia Commonwealth University Medical Center, Richmond (J.R.-C.).; 13 Beth Israel Deaconess Medical Center, Boston, MA (A.T.).; 14 Interventional Neuroradiology, University of California San Francisco, San Francisco (S.W.H.).; 15 Cooper University Hospital, Camden (B.T.J.).

**Keywords:** angiography, intracranial aneurysm, middle cerebral artery, retreatment, stent

## Abstract

Stent-assisted coil embolization using the new generation Neuroform Atlas Stent System has shown promising safety and efficacy. The primary study results of the anterior circulation aneurysm cohort of the treatment of wide-neck, saccular, intracranial, aneurysms with the Neuroform Atlas Stent System (ATLAS trial [Safety and Effectiveness of the Treatment of Wide Neck, Saccular Intracranial Aneurysms With the Neuroform Atlas Stent System]) are presented.

**Methods:**

ATLAS IDE trial (Investigational Device Exemption) is a prospective, multicenter, single-arm, open-label study of wide-neck (neck ≥4 mm or dome-to-neck ratio <2) intracranial aneurysms in the anterior circulation treated with the Neuroform Atlas Stent and approved coils. The primary efficacy end point was complete aneurysm occlusion (Raymond-Roy class 1) on 12-month angiography, in the absence of retreatment or parent artery stenosis (>50%) at the target location. The primary safety end point was any major stroke or ipsilateral stroke or neurological death within 12 months. Adjudication of the primary end points was performed by an independent Imaging Core Laboratory and the Clinical Events Committee.

**Results:**

A total of 182 patients with wide-neck anterior circulation aneurysms at 25 US centers were enrolled. The mean age was 60.3±11.4 years, 73.1% (133/182) women, and 80.8% (147/182) white. Mean aneurysm size was 6.1±2.2 mm, mean neck width was 4.1±1.2 mm, and mean dome-to-neck ratio was 1.2±0.3. The most frequent aneurysm locations were the anterior communicating artery (64/182, 35.2%), internal carotid artery ophthalmic artery segment (29/182, 15.9%), and middle cerebral artery bifurcation (27/182, 14.8%). Stents were placed in the anticipated anatomic location in all patients. The study met both primary safety and efficacy end points. The composite primary efficacy end point of complete aneurysm occlusion (Raymond-Roy 1) without parent artery stenosis or aneurysm retreatment was achieved in 84.7% (95% CI, 78.6%–90.9%) of patients. Overall, 4.4% (8/182, 95% CI, 1.9%–8.5%) of patients experienced a primary safety end point of major ipsilateral stroke or neurological death.

**Conclusions:**

In the ATLAS IDE anterior circulation aneurysm cohort premarket approval study, the Neuroform Atlas stent with adjunctive coiling met the primary end points and demonstrated high rates of long-term complete aneurysm occlusion at 12 months, with 100% technical success and <5% morbidity.

**Registration:**

URL: https://www.clinicaltrials.gov. Unique identifier: NCT02340585.

## Abstract 2

Supplemental Digital Content is available in the text.

Stent-assisted coil embolization for wide-neck distal aneurysm is a well-established endovascular transarterial treatment approach for cerebral aneurysms.^1–4^ Currently approved endovascular approaches include the use of detachable coils, intraluminal flow diverters, and more recently intrasaccular flow disruptors.^5–7^ However, a considerable proportion of clinically encountered cerebral aneurysms are wide neck (≥4 mm), distally located, and small in size (dome-to-neck ratio <2), requiring miniaturizing of the access and supportive balloon and microstent assistive coiling devices to achieve safer endovascular therapy. Technological advances in stents have allowed for the endovascular treatment of aneurysms that in the past would have resulted in a poor outcome or would have not been possible due to morphological characteristics of the aneurysm.^4^ In comparison to balloon-assisted coiling, stent-assisted coiling of wide-neck aneurysms may yield lower rates of retreatment and higher rates of aneurysm obliteration and progression of occlusion at follow-up, with a similar morbidity rate.^8^

The newest generation Neuroform Atlas (Atlas) stent represents a recent advance in cerebral laser-cut microstents to scaffold and support detachable intrasaccular coils, preventing coil protrusion while providing flow redirection with low rate of diversion.^4,9–11^ First approved >17 years ago, the original Neuroform stent has the longest-standing proven safety and efficacy in Stent-assisted coil aneurysm treatment worldwide.^1–4^ Building on the extensive clinical experience of its predecessor, the new generation of the Neuroform stent is designed for improved delivery via the same microcatheter used for coil embolization, scaled down to an internal diameter of 0.0165 inches (versus the 0.027-inch Neuroform EZ). Additionally, new features enhance ease of use such as a hybrid cell design with flaring of the proximal and distal portion of the Atlas stent, providing superior wall apposition and robust coil scaffolding.^9^

The Atlas microstent has previously shown excellent efficacy and safety results in a Food and Drug Administration (FDA) HDE study (Humanitarian Device Exemption) cohort of 30 patients in the anterior and posterior circulation.^9^ On May 16, 2019, FDA granted Pre-Market Approval of the Atlas stent in the treatment of wide-neck aneurysms in the anterior circulation of the neurovasculature. An independently powered cohort of posterior circulation patients stopped enrollment early for predictive success of primary safety and efficacy end points and is under review with FDA.

## Methods

### Study Design

The ATLAS IDE trial (Safety and Effectiveness of the Treatment of Wide Neck, Saccular Intracranial Aneurysms With the Neuroform Atlas Stent System Investigational Device Exemption) is a prospective, multicenter, open-label, nonrandomized single-arm study conducted at 25 medical centers in the United States. Eligible patients evaluated by the treating physician who met inclusion and exclusion criteria presenting with unruptured wide-necked, saccular, intracranial aneurysms of the anterior circulation meeting eligibility for stent-assisted coiling were treated using the Neuroform Atlas Stent System. Primary safety and efficacy end points were evaluated in a modified intention-to-treat cohort, defined as patients who signed the informed consent form and in whom a procedure was attempted. Patients not included in the analysis were patients who did not undergo an endovascular procedure and therefore were not treated. These patients were considered as enrolled but not treated. To ensure consistency and accuracy, angiographic end points were adjudicated by an independent Imaging Core Lab and safety end points by an independent Clinical Events Committee.

An IDE study protocol was approved by institutional review boards at each center, and patients provided written informed consent before participation in the trial. All data were entered into a Health Insurance Portability and Accountability Act-compliant electronic data capture system and monitored by the sponsor and a contract research organization. The data supporting the findings of this study are available from the corresponding author on reasonable request. The national principal investigators (Drs Zaidat and Jankowitz) had full access to the data and statistical analysis support. The sponsor approved the final version of the article for publication.

### Patient Enrollment

Patient identification, recruitment, and consecutive enrollment were performed by Clinical Investigators and designated research staff at each enrolling center. Key criteria for participation in the study included (1) being between 18 and 80 years old; (2) having an intracranial aneurysm within the anterior circulation (excluding the petrous ICA to the superior hypophyseal ICA region); and (3) having a wide-necked intracranial aneurysm (neck ≥4 mm or dome-to-neck ratio <2) and parent vessel diameter between 2.0 and 4.5 mm. Patients were excluded who had (1) multiple untreated intracranial aneurysms; (2) an acutely ruptured aneurysm within 14 days of enrollment; (3) premorbid modified Rankin Scale (mRS) score of ≥4 or premorbid Hunt and Hess score ≥3; (4) underlying parent artery atherosclerosis; (5) cerebral vascular malformation or intracranial mass; (6) prior treatment of the same aneurysm with stent-assisted coil embolization; (7) Moya-Moya disease; (8) absolute contraindication to angiography or antiplatelet therapy; among other study-specific criteria. Full detailed study enrollment criteria are available in Table I in the Data Supplement.

### Procedure

Details of the procedure were previously described in the primary results of the Atlas HDE study.^8^ Briefly, patients undergoing treatment received daily oral aspirin (81-325 mg) and clopidogrel (75 mg) for ≥5 days before the implant procedure, including the day of the procedure. Platelet function assays were not required per protocol. All procedures were performed under general anesthesia, and anticoagulation was managed per study site standard of care with a recommended activated clotting time of 250 to 300 seconds during the procedure. A transfemoral percutaneous approach using a guide catheter selected by the neurointerventionalist was used to obtain access to the target aneurysm. Microcatheters used were either an Excelsior SL-10 or XT-17 (Stryker Neurovascular, Fremont, CA) over a 0.014-inch microguidewire. The ATLAS microstent was then deployed as per the standard technique and coils were placed in the aneurysm during the same procedure. Post-treatment dual antiplatelet regimen was maintained for at least 3 months following stent implantation.

### Follow-Up

Follow-up clinical examinations for enrolled and treated patients were performed throughout hospitalization up to the day of discharge and at 2, 6, and 12 months postprocedure. Data were collected on primary and secondary end points, and additional assessments were conducted, including National Institute of Health Stroke Scale, mRS, Hunt and Hess if previous aneurysm rupture and evidence of subarachnoid hemorrhage, antiplatelet compliance, adverse events, and a validated quality of life EuroQol-5 Dimension-3 Levels (EQ-5D-3L) questionnaire. At the 12-month follow-up, digital subtraction angiography was required to assess the rate of complete aneurysm occlusion.

### Primary Study Outcomes

#### Efficacy

The primary efficacy end point was the rate of complete aneurysm occlusion, defined as 100% occlusion (Raymond-Roy [RR] class 1)^12,13^ of the target aneurysm on the 12-month follow-up angiogram with no aneurysm retreatment and no parent artery stenosis (>50%) at the target location. All images were reviewed, and occlusion rates adjudicated by an independent imaging core lab (University of California San Francisco Interventional Radiology Research Laboratory, San Francisco, CA) blinded to study site operator assessment of outcomes and clinical data. The primary efficacy success threshold was set at >50%. Historical data from stented patients in the MAPS trial (Matrix and Platinum Science),^14^ and a meta-analysis of contemporary peer-reviewed published literature served as the basis for establishing the efficacy performance goal.

#### Safety

The primary safety end point was the rate of any major ipsilateral stroke or neurological death within 12 months following the procedure. An ipsilateral stroke was defined as an acute episode of focal or global neurological dysfunction due to brain or retinal infarction or due to an intracranial hemorrhage inclusive of subarachnoid, intraventricular, or intraparenchymal hemorrhage, occurring in the same hemisphere as the target aneurysm. A major ipsilateral stroke was defined as an ipsilateral stroke with an increase of ≥4 points on the National Institute of Health Stroke Scale assessment at 24 hours after symptoms onset. A Clinical Events Committee evaluated any serious device-related events and prespecified safety event end points. A threshold of <20% was set for the anterior circulation cohort in the ATLAS trial. Safety performance goals were established using data extracted from the MAPS trial^14^ wide-neck aneurysm patient cohort along with published rates of procedural and long-term morbidity and mortality for embolic coiling, balloon-assisted embolic coiling, or stent-assisted coiling.

### Secondary Outcomes

#### Efficacy

Secondary efficacy end points included procedural technical success (ie, proportion of patients in whom the Neuroform Atlas was successfully delivered and deployed at the target neck location), target aneurysm retreatment, rates of aneurysm recanalization and occlusion, and incidence of parent artery stenosis (>50% stenosis).

#### Safety

The secondary safety end point was the percentage of patients experiencing ≥1 of the following serious adverse events: worsening major ipsilateral stroke as measured by National Institute of Health Stroke Scale, device-related serious adverse events, rate of subarachnoid hemorrhage, and rate of target aneurysm rupture.

### Statistical Analysis

Descriptive statistics were compiled on baseline variables, procedural characteristics, and end points. Continuous and ordinal variables are summarized with mean, SD, median, and interquartile range. Median and interquartile range are reported when distribution of a variable is visually skewed from normal distribution. Percentages, numerators, and denominators are presented for categorical and binary variables.

The proportion of subjects who met the primary end points are compared with performance goals using the 1-sided Fisher exact test, with a significance level of α=0.025. The performance goals were determined a priori based on a meta-analysis, as well as regulatory and medical considerations. The proportion of safety failure was hypothesized to be ≤0.20 (safety performance goal), and the proportion of efficacy success was ≥0.50 (efficacy performance goal).

For the efficacy end point, multiple imputation was performed to fill in missing data. A logistic regression model was fit using age, sex, and race as the independent variable, with probability of success as the dependent variable. The fill-in process was repeated five times. The pooled proportion of successful subjects was compared with the a priori performance goal. For sensitivity analyses, efficacy results were computed by using complete case only and by using worst-case analysis. All analyses were performed using SAS, version 9.4 (Cary, NC). *P* values are unadjusted for multiplicity.

## Results

Between June 2015 and October 2016, 201 patients with anterior circulation brain aneurysms were enrolled across 25 US centers, of which 182 (90.5%) were in the modified intention-to-treat cohort (Figure). Twelve-month follow-up on patients was completed in October 2017. Mean patient age was 60.3±11.4 years, with 73.1% female and 80.8% white (Table 1). The most frequent comorbidities were hypertension (74.2%) and hyperlipidemia (54.4%), whereas over half of all patients were current or past cigarette users. Nineteen (10.4%) patients reported a history of multiple aneurysms, and 28 (15.4%) had a previous hemorrhagic stroke. A total of 95.6% of patients had a baseline mRS score of ≤2. Overall, 22 (12.1%) patients experienced previous rupture of target aneurysms: 16 (72.7%) treated with coiling, 4.5% with balloon-assisted coiling, and 22.7% by other means.

**Table 1.**
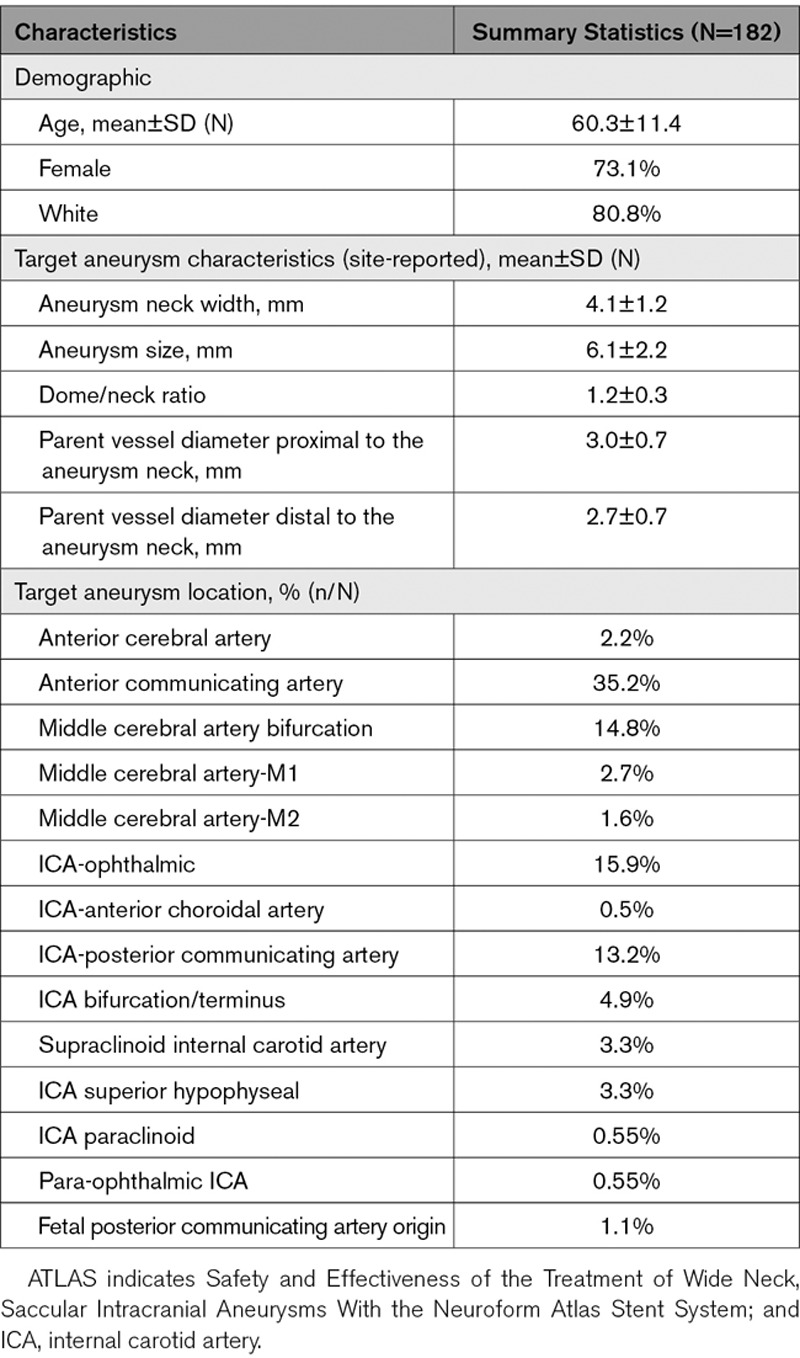
Baseline Characteristics of the ATLAS Trial Anterior Circulation Cohort

**Figure.**
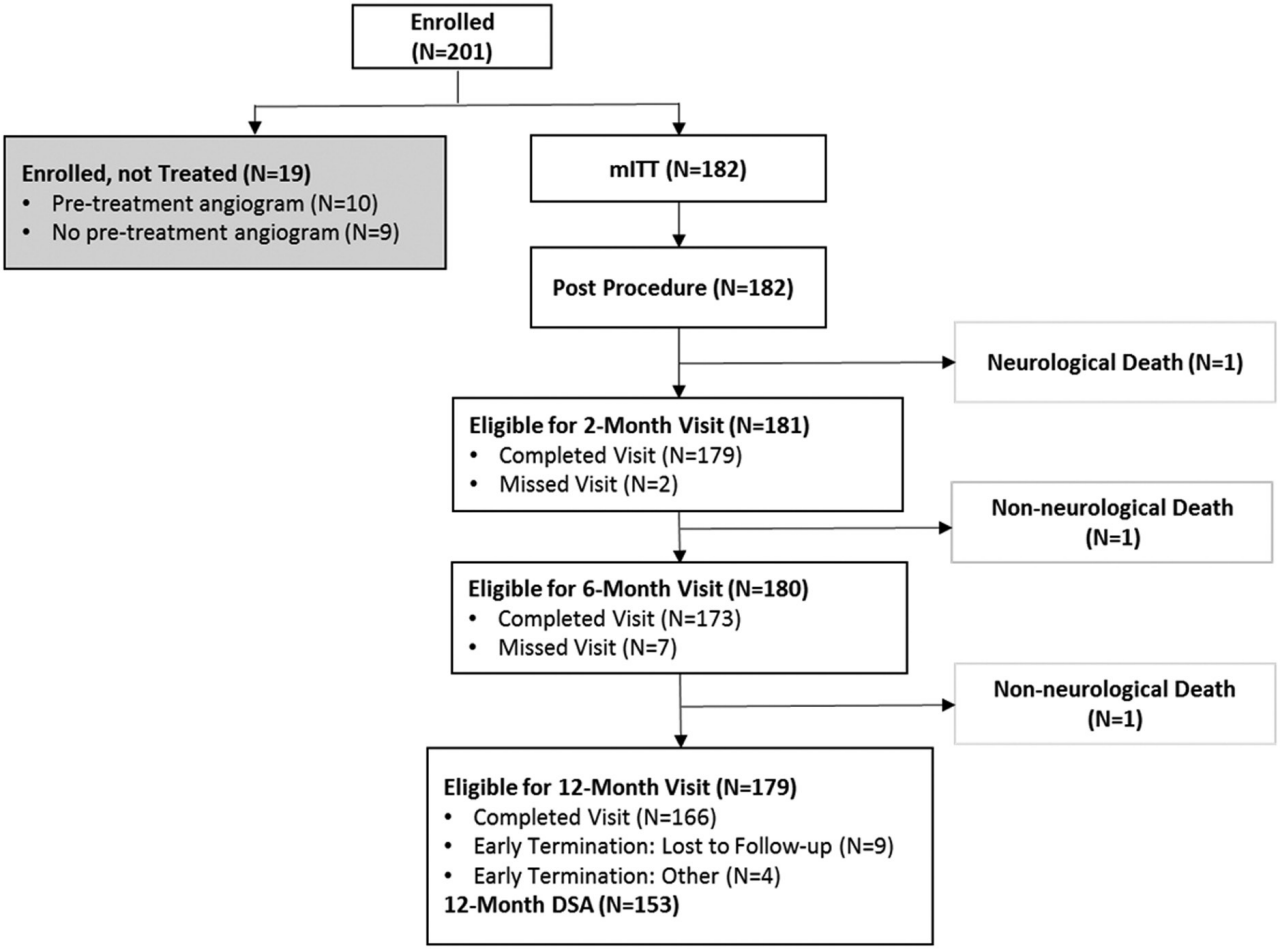
**ATLAS (Safety and Effectiveness of the Treatment of Wide Neck, Saccular Intracranial Aneurysms With the Neuroform Atlas Stent System) patient flow diagram.** mITT indicates modified intention-to-treat

Target aneurysm characteristics are summarized in Table 1. Briefly, the mean aneurysm size was 6.1±2.2 mm, mean neck width was 4.1±1.2 mm, and mean dome/neck ratio was 1.2±0.3. The most common aneurysm locations were the anterior communicating artery (35.2%), ophthalmic segment of the internal carotid artery (15.9%), middle cerebral artery bifurcation (14.8%), and the internal carotid artery (ICA)-posterior communicating artery segment (13.2%).

### Intraprocedural and Postprocedural Results

The protocol allowed for the implantation of up to 2 Atlas stents in a single procedure. Procedural success was achieved in 100% of patients, with implantation of 1 stent occurring in 153 of 182 (84.1%) patients, and 2 stents in 29 (15.9%) patients. Implant success was achieved in 93.0% of attempted devices. The median number of coils was 5 (interquartile range, 3–7), and median procedure time, defined as the time from first puncture to wound closure, was 101.0 (interquartile range 80.0–137.0) minutes. Angiographic imaging was adjudicated by an independent core laboratory for 180 patients, with complete occlusion postprocedure (RR, 1) 77.8%, residual aneurysm neck filling (RR, 2) 15.6%, and residual aneurysm dome filling (RR, 3) 6.7%. Imaging was not available for 2 patients.

### Primary End Points

#### Efficacy

Of the 166 patients who completed the 12-month follow-up, 153 patients had Digital Subtraction Angiography (DSA) results available (Figure, Table 2). Complete occlusion, per the imaging core laboratory, was achieved in 88.2% (95% CI, 82.0–92.9) of patients, with parent artery stenosis >50% occurring in 1.3% (95% CI, 0.2–4.6). Retreatment occurred in 7 of 182 patients (3.8% [95% CI, 1.6–7.8]), of which 4 patients (2.2%) had complete occlusion intraprocedurally, 2 (1.1%) had preplanned staged procedures, and 1 (0.5%) had residual neck. The primary efficacy composite end point of complete occlusion in the absence of retreatment or significant parent artery stenosis was achieved in 84.7% (95% CI, 78.6–90.9; *P*<0.001).

**Table 2.**
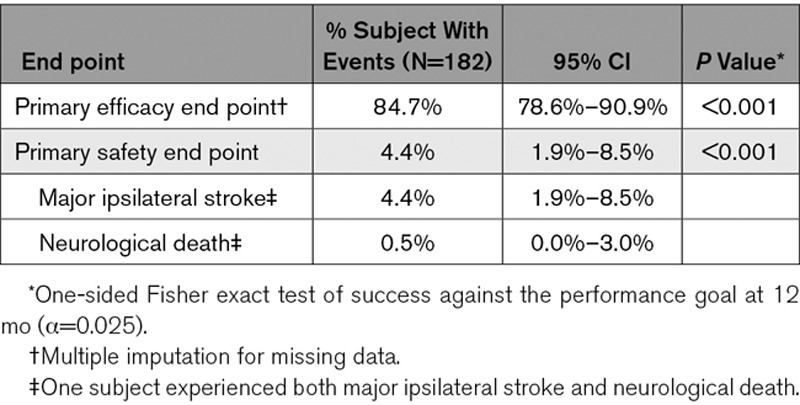
Primary Safety and Efficacy End Points

#### Safety

Incidence of the primary safety composite end point (major ipsilateral stroke and/or neurological death) for all 182 patients was 4.4% (8/182 [95% CI, 1.9–8.5]; *P*<0.001; Table 2). Eight patients had a major ipsilateral stroke (4.4% [95% CI, 1.9–8.5]), with one also experiencing neurological death (0.5% [95% CI, 0.0–3.0]). Four patients recovered with no residual deficit on subsequent follow-up and with 4 of 182 (2.2%) patients having symptomatic long-term outcome or death.

### Secondary End Points

#### Efficacy

Secondary end points were evaluated at 12-month follow-up, and the results are summarized in Table 3. Of 153 subjects, 135 had RR, 1 occlusion of the target aneurysm (88.2% [95% CI, 82.0–92.9]) at 12-month DSA, 12 had RR, 2 (7.8% [95% CI, 4.1–13.3]), and six had RR, 3 (3.9% [95% CI, 1.5–8.3]). One hundred forty-seven subjects had combined RR, 1 and 2 (96.1% [95% CI, 91.7–98.5]). Overall, out of 153 subjects, 145 (94.8%) had the same (60.1%) or improved (34.6%) occlusion status of their target aneurysms at 12 months compared to immediate postprocedure RR scores, whereas 8 (5.2%) subjects had worse occlusion status compared to postprocedure RR scores. Clinically, 152 of 166 (91.6%) patients had an mRS score of 0 to 1, whereas 157 (94.6%) had an mRS score of 0 to 2.

**Table 3.**
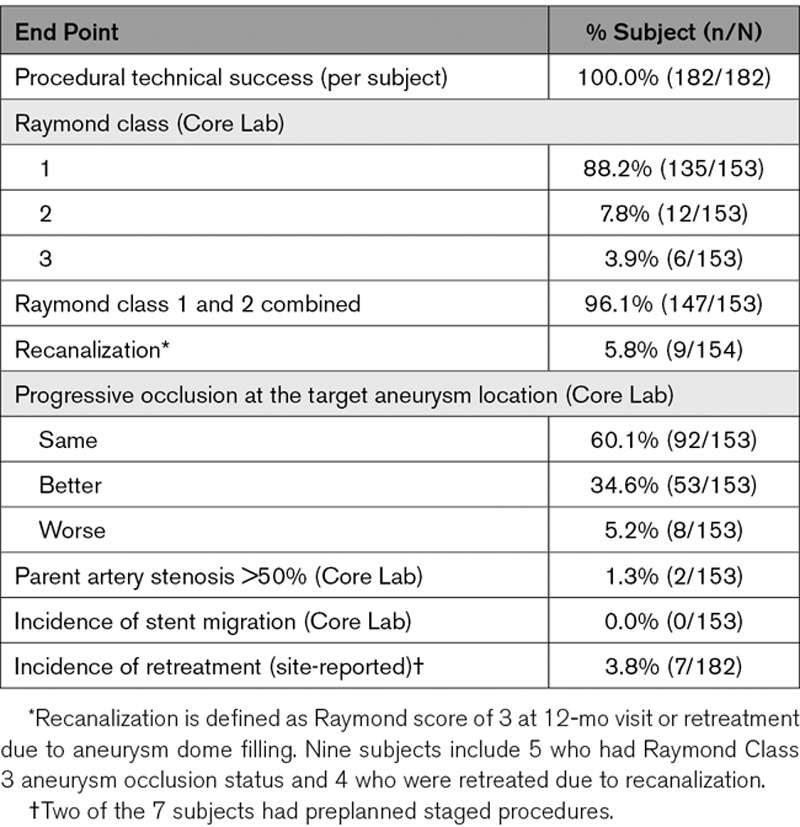
Secondary Efficacy End Points at 12-Month Follow-Up

#### Safety

Subarachnoid hemorrhage and aneurysm rupture were secondary end points adjudicated by the Clinical Events Committee (Table 4). Seven subjects experienced the secondary safety end point of subarachnoid hemorrhage or rupture. Two of these subjects also had major ipsilateral stroke or death adjudicated as primary end points. There were 5 patients that experienced either a subarachnoid hemorrhage or aneurysm rupture. Four patients experienced both and 1 patient only had subarachnoid hemorrhage. All 5 of these patients returned for their 12-month visit with mRS ≤1.

**Table 4.**
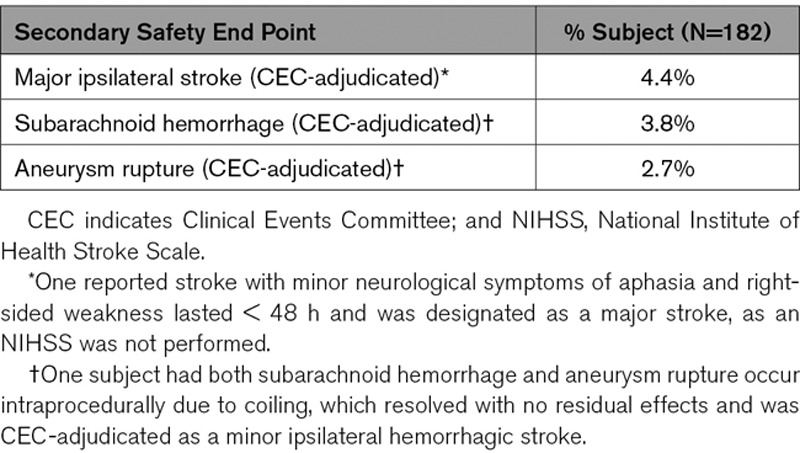
Secondary Safety End Points Through 12-Month Follow-Up

Summary of the 9 events in 8 subjects as adjudicated by the Clinical Events Committee is shown in Table II in the Data Supplement.

## Discussion

The pivotal ATLAS IDE study is the largest of its kind, with an anterior circulation cohort of 182 patients across 25 US centers. The Neuroform Atlas Stent System successfully occluded wide-neck intracranial aneurysms in the anterior circulation without stenosis or retreatment in the vast majority of patients (84.7%), and the Clinical Events Committee–adjudicated safety end point rate was 4.4%. Study success was achieved through both primary efficacy and safety end points being met based on evaluation of modified intention-to-treat cohort patients at 12-month follow-up. Based on the accomplishment of the ATLAS IDE trial in demonstrating substantial safety and efficacy in the large anterior circulation cerebral aneurysm, the FDA granted Pre-Market Approval of the device in May 2019. An independently powered cohort of posterior circulation patients stopped early for predictive success of primary safety and efficacy end points and is under review with FDA.

The ATLAS IDE anterior cohort results are consistent with other recent studies of patients treated with Atlas stent-assisted coil embolization for wide-necked distal aneurysms. These studies, however, were limited in scope as they analyzed smaller cohorts of patients. A retrospective trial in Germany studied 37 patients treated with Atlas between 2014 and 2018 and showed an immediate complete aneurysm occlusion rate of 83.8%, 6-month angiographic follow-up occlusion rate of 80.8%, and 2.7% permanent neurological morbidity.^11^ Another study in Italy evaluated 113 patients with wide-necked cerebral aneurysms treated with Atlas and showed an immediate occlusion rate of 88%, 82% at 12-month follow-up, whereas RR scores of 1 or 2 were 95% at 12-month follow-up. Overall complication rate was 6.2% in that study.^15^ Following Atlas HDE approval in the United States, a single-center retrospective study of 58 patients with 76 Atlas stents in >70% wide-necked distal aneurysms demonstrated an immediate occlusion rate of 70.7%, overall complication rate of 6.9%, and 0% permanent neurological deficit on discharge or day 11 follow-up.^16^ All 3 studies demonstrated 100% technical success and echo the efficacy and safety findings of ATLAS IDE. Additionally, the Atlas stent design has been shown to be feasible for Y-stent–assisted and X-stent–assisted coiling as demonstrated in the recent study of 55 patients with wide-neck bifurcation aneurysms, achieving 87% rate of complete occlusion at a mean follow-up of 16 months, with 12.7% rate of overall peri-procedural complications.^17^

The Atlas stent constitutes a significant advance in open-cell laser-cut stents. With its improved technical attributes, the low-profile Atlas stent is compatible with a highly navigable 0.0165” ID microcatheter. The Atlas stent employs a hybrid cell design – utilizing primarily open-cell features that provide conformability with a proximal closed-cell feature to enhance stability.^9^ In comparison to devices such as the Low-Profile Visualized Intraluminal Support Junior Device (LVIS Jr), there are inherent technical differences between open-cell laser cut and woven braided stents. Laser-cut stents provide a more precise landing zone with less foreshortening, whereas braided stents provide full-length radiopacity. Open-cell stents provide better wall apposition while woven stents provide resheathability.

Clinically, Atlas and LVIS Jr also appear to have distinct efficacy and safety profiles. Although several studies have looked at the clinical and angiographic results of aneurysms treated with the LVIS Jr stents, few have compared it with Atlas. Gross et al^10^ evaluated concurrent experiences with both Atlas and LVIS Jr stents, and their findings show a significantly higher rate of immediate complete occlusion using Atlas (57% versus 41%, *P*=0.03). Long-term angiographic follow-up showed significantly higher rates of RR, 1 and 2 in the Atlas stent group (100% versus 81%, *P*=0.04) with a lower rate of in-stent stenosis (0% versus 19%, *P*=0.04). These differences between devices were demonstrated despite similar patient cohorts as defined at baseline by age, sex, aneurysm location, and aneurysm size.

These results are consistent when comparing pivotal the ATLAS IDE trial results with pivotal LVIS Jr IDE findings,^18^ which showed a numerically higher rate of complete target aneurysm occlusion with Atlas versus LVIS Jr stent-assisted coiling (88.2% versus 79.1%). Rates of retreatment were similar between devices, with Atlas at 3.8% and LVIS Jr 3.9%. Numerically lower rates of stent thrombosis (1.6% Atlas versus 3.3% LVIS Jr) and mortality (0.5% Atlas versus 1.7% LVIS Jr) were also noted for the Atlas stent in comparison with LVIS Jr.

Another pivotal IDE study of treatment of wide-neck aneurysms is the WEB-IT (Woven EndoBridge Intrasaccular Therapy).^7^ Rates of retreatment were numerically greater with the Web device as compared to Atlas (9.8% versus 3.8%). Complete aneurysm occlusion at 12-month angiographic follow-up was numerically higher in Atlas versus WEB (88.2% versus 53.8%), whereas mortality rates were lower in Atlas (0.5% versus 0.7%).

### Study Limitation

The pivotal ATLAS IDE study is a single-arm study; however, its design characteristics such as independent central imaging core lab and independent clinical event committee were embedded to reduce bias. Use of flow diverters for aneurysm treatment has also increased recently; thus, comparison with older aneurysm treatment studies may be limited by different patient selection paradigms in contemporary practice.

### Conclusions

The pivotal ATLAS IDE study demonstrates the Neuroform Atlas Stent System is efficacious and has a favorable safety profile for patients with wide-neck anterior circulation aneurysms. In conjunction with commercially available coils, Atlas successfully occluded target aneurysms without clinically significant parent artery stenosis or retreatment in most patients. Treatment with Atlas is also associated with a low rate of serious neurological events and mortality.

## Sources of Funding

The ATLAS trial (Safety and Effectiveness of the Treatment of Wide Neck, Saccular Intracranial Aneurysms With the Neuroform Atlas Stent System) was funded by Stryker Neurovascular.

## Disclosures

Drs Zaidat, Hanel, Jovin, Gupta, Siddiqui, Hetts, Sauvageau, Frei, Malek, and Jankowitz reports Research Grant, Speaker, Consultant, Personal fees from Stryker. Dr Hanel is a consultant for Medtronic, Microvention, Balt, eLum, Three Rivers Medical, Cerebrotech, RIST, Blink TBI, and Imperative, shareholder in eLum, Three Rivers Medical, Cerebrotech, RIST, Blink TBI, Endostream, InNeuroCo, Serenity, and Scientia, and on SAB for eLum, and Three Rivers Medical, and other disclosure for Phenox. Dr Jovin is an investor/advisor for Route92, Vizai, FreeOx, Blockade Medical, Corindus, on the DSMB for Cerenovus, receives personal fees from Cerenovus and Medtronic, and is a consultant for Medtronic. Dr Gupta is a consultant and receives personal fees from Cerenovus. Dr Malek is a cofounder/investor for CereVasc LLC and holds a patent US9095420B2. Dr Gabor receives personal fees from Microvention, Medtronic, and DynaMed. Dr Siddiqui reports grants from Coinvestigator: NIH/NINDS 1R01NS091075 Virtual Intervention of Intracranial Aneurysms; Role: Co-Principal Investigator NIH-NINDS R21 NS109575-01 Optimizing Approaches to Endovascular Therapy of Acute Ischemic Stroke, personal fees from Adona Medical, Inc, Amnis Therapeutics, BlinkTBI, Inc, Boston Scientific Corp (for purchase of Claret Medical), Buffalo Technology Partners, Inc, Cardinal Consultants, LLC, Cerebrotech Medical Systems, Inc, Cognition Medical, Endostream Medical, Ltd, Imperative Care, Inc, International Medical Distribution Partners, Neurovascular Diagnostics, Inc, Q’Apel Medical, Inc, Rebound Therapeutics Corp (Purchased 2019 by Integra Lifesciences, Corp), Rist Neurovascular, Inc, Sense Diagnostics, Inc, Serenity Medical, Inc, Silk Road Medical, Spinnaker Medical, Inc, StimMed, Synchron, Three Rivers Medical, Inc, Vastrax, LLC, VICIS, Inc, Viseon, Inc, personal fees from Amnis Therapeutics, Boston Scientific, Canon Medical Systems USA, Inc, Cerebrotech Medical Systems, Inc, Cerenovus, Corindus, Inc, Endostream Medical, Ltd, Imperative Care, Inc, Integra LifeSciences Corp, Medtronic, MicroVention, Minnetronix Neuro, Inc, Northwest University, Penumbra, Q’Apel Medical, Inc, Rapid Medical, Rebound Therapeutics Corp, Serenity Medical, Inc, Silk Road Medical, StimMed, Stryker, Three Rivers Medical, Inc, VasSol, W.L. Gore & Associates, and personal fees from Cerenovus; Medtronic; MicroVention; consultant fees for opinion on the design of clinical trials. The other authors report no conflicts.

## Supplementary Material



## Appendix

Lyerly Neurosurgery-Baptist: Ricardo Hanel (PI), Eric Sauvageau (Sub-I), Amin Nima Aghaebrahim (Sub-I); Mercy St. Vincent: Osama Zaidat (PI), Eugene Lin (Sub-I); University of Pittsburgh: Ashutosh Jadhav (PI and past Sub-I), Bradley Gross (Sub-I), Brian Jankowitz (past Sub-I and past PI), Andrew Ducruet (past Sub-I), David Panczkowski (past Sub-I), Hazem Shoirah (past Sub-I), Alhamza Al-Bayati (past Sub-I), Amin Aghaebrahim (past Sub-I), Tudor Jovin (past Sub-I), Greg Weiner (past Sub-I), Cynthia Kenmuir (past Sub-I), Prasanna Tadi (past Sub-I), Gregory Walker (past Sub-I); WellStar Research Institute: Ahmad Khaldi (PI), Rishi Gupta (Sub-I), K. Johnson (past Sub-I); RIA/Swedish: Don Frei (Current PI and past Sub-I), Richard Bellon (Sub-I), Benjamin Atchie (Sub-I), Ian Kaminsky (Sub-I), David Loy (Past PI and Sub-I), Dan Huddle (past Sub-I); Tufts Medical Center: Adel Malek (PI); Cleveland Clinic Foundation: Gabor Toth (PI), Mark Bain (Sub-I), Peter Rasmussen (Sub-I), M. Shazam Hussain (Sub-I), Nina Moore (Sub-I), Thomas Masaryk (Sub-I), Mohamed Elgabaly (Sub-I), Russell Cerejo (past Sub-I), Julian Hardman (past Sub-I), Seby John (past Sub-I), Andrew Bauer (past Sub-I), Jenny Peih-Chir Tsai (past Sub-I); SUNY Buffalo: Adnan Siddiqui (PI), Elad Levy (Sub-I), Kenneth Snyder (Sub-I), Jason Davies (Sub-I); Beth Israel Deaconess: Ajith Thomas (PI), Christopher Ogilvy (Sub-I); Virginia Commonwealth: John Reavey-Cantwell (PI), Dennis Rivet (Co-PI); Cedars Sinai Medical Center: Michael Alexander (PI), Franklin Moser (Sub-I), Marcel Maya (Sub-I), Michael Schiraldi (Sub-I), Paula Eboli (past Sub-I); Johns Hopkins University: Justin Caplan (Current PI), Bowen Jiang (Sub-I), Matthew Bender (Sub-I), Geoffrey Colby (past PI); Christiana Care Health Services: Sudhakar Satti (PI), Thinesh Sivapatham (Sub-I); Hospital of the University of PA: David Kung (Current PI and past Sub-I), Bryan Pukenas (Sub-I), Robert Hurst (Sub-I), Michelle J. Smith (past PI); Univ of Massachusetts: Ajit Puri (PI), Francesco Massari (Sub-I), David Rex (past Sub-I),; University of Kentucky: Justin Fraser (Current PI and past Sub-I), Stephen Grupke (Sub-I), Abdulnasser Alhajeri (past PI); Houston Methodist Hospital: Richard Klucznik (PI), Orlando Diaz (Sub-I), Gavin Britz (Sub-I), Yi Zhan (Sub-I); Medical University of South Carolina: Alejandro Spiotta (Current PI and past Sub-I), Jonathan Lena (Sub-I), Aquilla Turk (past PI), Mohamad Chaudry (past Sub-I), Kyle Fargen (past Sub-I), Raymond Turner (past Sub-I); Baylor College of Medicine: Peter Kan (PI), Edward Duckworth (past Sub-I); Los Robles Regional: Muhammad Asif Taqi (PI), Samuel Hou (past Sub-I); Methodist Healthcare Memphis: Adam S. Arthur (PI), Lucas Elijovich (Sub-I), Daniel Hoit (Sub-I), Christopher Nickele (Sub-I), Jay Vachhani (past Sub-I), Vinodh Thomas Doss (past Sub-I); Rush University: Richard Crowley (Current PI and past Sub-I), Demetrius Lopes (past PI), Michael Chen (past Sub-I); Harborview Medical Center: Danial Hallam (PI), Basavaraj Ghodke (Sub-I), Louis Kim (Sub-I); SSM DePaul Health Center: Richard Callison (PI), Amer Alshekhlee (Sub-I), Sushant Kale (past Sub-I); Vanderbilt University: Michael Froehler (PI), Matt Fusco (Sub-I), Rohan Chitale (Sub-I).
