# Supplementary material for: Pivotal Trial of the Neuroform Atlas Stent for Treatment of Anterior Circulation Aneurysms: One-Year Outcomes
Source: Stroke. 2020 Jun 17;51(7):2087–94. doi: 10.1161/STROKEAHA.119.028418 (PMC7306258; doi:10.1161/STROKEAHA.119.028418)
Supplement: Supplementary file 1 [file str-51-2087-s001.pdf]

SUPPLEMENTAL MATERIAL

Supplementary Table I: ATLAS Anterior Circulation Cohort Inclusion and Exclusion Criteria

**Study Inclusion Criteria**
1. Subject is between 18 and 80 years of age
2. Subject has a documented, wide neck (neck ≥ 4 mm or a dome-to-neck ratio < 2), anterior circulation, intracranial, saccular aneurysm arising from a parent vessel with a diameter of ≥ 2 mm and ≤ 4.5 mm, which will be treated with bare metal coils
3. Subject or legal representative is willing and able to provide informed consent
4. Subject is willing and able to comply with protocol follow up requirements
**Study Exclusion Criteria**
1. Subject has known multiple untreated cerebral aneurysms, other than non-target blister aneurysm, infundibulum, or aneurysm measuring < 3 mm for each of three dimensions assessed (height, width, and depth) that will not require treatment during the study period
2. Subject has a target lesion that is a blister aneurysm, infundibulum, or aneurysm measuring < 3 mm for each of three dimensions assessed (height, width, and depth)
3. Subject has a target aneurysm that will require an Investigator to intentionally leave a neck remnant in order to preserve blood flow in a bifurcation or branch
4. Subject has undergone coiling or stenting of a non-target intracranial aneurysm within 30 days prior to study treatment
5. Subject has a target aneurysm in the anterior circulation proximal to the superior hypophyseal ICA
6. Subject has acute target aneurysm rupture less than 14 days prior to study treatment
7. Subject has a Hunt and Hess score ≥ 3 or a premorbid mRS score ≥ 4
8. Subject has an admission platelet count of < 50,000, any known coagulopathy, or an International Normalized Ratio (INR) > 3.0 without oral anticoagulation therapy
9. Subject has a known absolute contraindication to angiography
10. Subject has evidence of active cancer, terminal illness, or any condition which, in the opinion of the treating physician, would/could prevent the subject from completing the study (e.g., a high risk of embolic stroke, atrial fibrillation, co-morbidities, psychiatric disorders, substance abuse, major surgery ≤ 30 days pre-procedure, etc.)
11. Subject has a known absolute contraindication to the use of required study medications or agents (e.g., heparin, aspirin, clopidogrel, and radiographic contrast agents etc.)
12. Subject is female and is pregnant or intends to become pregnant during the study
13. Subject has Moya-Moya disease, arteriovenous malformation(s), arteriovenous fistula(e), intracranial tumor(s), or intracranial hematoma(s) (unrelated to target aneurysm)
14. Subject has significant atherosclerotic stenosis, significant vessel tortuosity, vasospasm refractory to medication, unfavorable aneurysm morphology or vessel anatomy, or some other condition(s) that, in the opinion of the treating physician, would/could prevent or interfere with access to the target aneurysm and/or successful deployment of the Neuroform Atlas Stent
15. Subject has had previous treatment (e.g., surgery, stenting) in the parent artery that, in the opinion of the treating physician, would/could prevent or interfere with successful use of the Neuroform Atlas Stent System and/or successful deployment of embolic coils
16. Subject has undergone previous stent-assisted coiling of the target aneurysm

Supplementary Table II: Neurological Events outcome and adjudication by the clinical event committee (CEC)

#	Neurological Event	Post procedure day	CEC Adjudication	Outcome
1	Hemorrhage: Intracranial and Subarachnoid Hemorrhage	8	Procedure/Device Related	Death-Day 9
2	Hemorrhage and Ischemic Stroke: Vessel perforation -Right MCA stroke, with SAH	0	Procedure/Device Related	Resolved with residual effects
3	Ischemic Stroke: Right MCA/ NIHSS 7	1	Procedure/Device Related	Resolved with residual effects
4	Ischemic Stroke: Left MCA	0	Procedure/Device Related	Resolved with residual effects
5	Ischemic Stroke: left MCA and ACA embolic process	2	Procedure/Device Related	Resolved without residual effects
6	Ischemic Stroke: Acute infarct in left corona radiata,	166	Device Related	Resolved with residual effects
7	Hemorrhagic: Cerebral Aneurysm rupture	0	Procedure/Device Related	Resolved with residual effects
8	Hemorrhagic: Aneurysm rupture due to coil packing.	0	Procedure Related	Resolved without residual effects
9	Hemorrhagic: SAH	0	Procedure/Device Related	Resolved without residual effects

[1] All 9 major ipsilateral stroke events (in 8 subjects) also met the secondary safety endpoint of 'new or worsening major ipsilateral stroke'

[2] Legend: SAH = Subarachnoid haemorrhage; MCA = Middle Cerebral Artery; ACA = Anterior Cerebral Artery; NIHSS=National Institute of Health Stroke Severity Scale

ATLAS Investigator Group (Sorted by number of patients recruited):

Lyerly Neurosurgery-Baptist (19)- Ricardo Hanel (PI), Eric Sauvageau (Sub-I), Amin Nima Aghaebrahim (Sub-I), Nancy Ebreo (CRC), Karen Bell (CRC), Lanaya Lewis (CRC);
Mercy St. Vincent (18) - Osama Zaidat (PI), Eugene Lin (Sub-I), Tina M. Steinhauser (CRC), Dee Tilley (CRC), Julie Goins-Whitmore (past CRC), Brandi Breseman (past CRC), Melissa A. Thomas (past CRC);
University of Pittsburgh (17) – Ashutosh Jadhav (PI and past Sub-I), Bradley Gross (Sub-I), Lisa Baxendell (CRC), Patricia Feineigle (CRC), Vicki Gilchrist (CRC), Brian Jankowitz (past Sub-I and past PI), Andrew Ducruet (past Sub-I), David Panczkowski (past Sub-I), Hazem Shoirah (past Sub-I), Alhamza Al-Bayati (past Sub-I), Amin Aghaebrahim (past Sub-I), Tudor Jovin (past Sub-I), Greg Weiner (past Sub-I), Cynthia Kenmuir (past Sub-I), Prasanna Tadi (past Sub-I), Gregory Walker (past Sub-I), Kelsea Haibach (past CRC), Carlynn Graves (past CRC), Yvonne Cannon (past CRC);
WellStar Research Institute (16) - Ahmad Khaldi (PI), Rishi Gupta (Sub-I), Marianne Bain (CRC), Laura Murphy (CRC), Andrew K. Johnson (past Sub-I), Barbara A. Foster (past CRC), Tasha Futch (past CRC), Portia Thomas (past CRC);
RIA/Swedish (14) - Don Frei (Current PI and past Sub-I), Richard Bellon (Sub-I), Benjamin Atchie (Sub-I), Ian Kaminsky (Sub-I), Lisa Kodis (CRC), Mark Talley (CRC), Tiffany Talley (CRC), Alex Edinger (CRC), David Loy (Past PI and Sub-I), Dan Huddle (past Sub-I), Michelle Lexin (past CRC), Brad Fasbinder (CRC), Alicia Drew (past CRC), Joanna Snead (past CRC), Ashley Bitner (past CRC), Sarah Weiss (past CRC), Nouara Sadaoui (past CRC);
Tufts Medical Center (12) - Adel Malek (PI), Emma Jost-Price (CRC), Keri Sullivan (CRC), Haley Huggins (past CRC), Lindsey Soll (past CRC), Sarah Gans (past CRC), Michelle Bettle (past CRC);
Cleveland Clinic Foundation (10) - Gabor Toth (PI), Mark Bain (Sub-I), Peter Rasmussen (Sub-I), M. Shazam Hussain (Sub-I), Nina Moore (Sub-I), Thomas Masaryk (Sub-I), Mohamed Elgabaly (Sub-I), Erin Bynum (CRC), Russell Cerejo (past Sub-I), Julian Hardman (past Sub-I), Seby John (past Sub-I), Andrew Bauer (past Sub-I), Erin Mayock (past CRC), Vikram Puvenna (past CRC), Jenny Peih-Chir Tsai (past Sub-I);
SUNY Buffalo (9) - Adnan Siddiqui (PI), Elad Levy (Sub-I), Kenneth Snyder (Sub-I), Jason Davies (Sub-I), Mary Hartney (CRC), Jonna Sakowski (CRC), Courtney Drozdowski (past CRC), Heather Ross (past CRC), Linda Bookhagen (past CRC);
Beth Israel Deaconess (7) - Ajith Thomas (PI), Christopher Ogilvy (Sub-I), Patricia Baum (CRC);
Virginia Commonwealth (7) - John Reavey-Cantwell (PI), Dennis Rivet (Co-PI), Charlotte Gilman (CRC);
Cedars Sinai Medical Center (6) - Michael Alexander (PI), Franklin Moser (Sub-I), Marcel Maya (Sub-I), Michael Schiraldi (Sub-I), Vicki Manoukian, MA (CRC), Paula Eboli (past Sub-I);
Johns Hopkins University (6) - Justin Caplan (Current PI), Bowen Jiang (Sub-I), Matthew Bender (Sub-I), Ellen Sheehan (CRC), Jessica Wollett (CRC), Geoffrey Colby (past PI), Lauren Dise (past CRC), Anna Bugaeva (past CRC), Barbara Michniewicz (past CRC), Thomas Hemmingson (past CRC);
Christiana Care Health Services (5) - Sudhakar Satti (PI), Thinesh Sivapatham (Sub-I), Robie Zent (CRC), Ann Marie Le Noir (CRC); Removed

Hospital of the University of PA (5) - David Kung (Current PI and past Sub-I), Bryan Pukenas (Sub-I), Robert Hurst (Sub-I), Timothy Prior (CRC), Whitney Sarchiapone (CRC), Yelena Gorelik (past CRC), Francis Quattrone (past CRC), Michelle J. Smith (past PI);
Univ of Massachusetts (4) -Ajit Puri (PI), Francesco Massari (Sub-I), Mary Howk (CRC), David Rex (past Sub-I), Kimberly Ty (past CRC), Jen Donham (past CRC), Wen Li (past CRC);
University of Kentucky (4) - Justin Fraser (Current PI and past Sub-I), Stephen Grupke (Sub-I), Jennifer Isaacs (CRC), Abdulnasser Alhajeri (past PI), Caroline Rodgers (past CRC); Linda Joyce McCown (past CRC);
Houston Methodist Hospital (4) - Richard Klucznik (PI), Orlando Diaz (Sub-I), Gavin Britz (Sub-I), Yi Zhang (Sub-I), Michelle Prystash (CRC), Vivian Escamilla (CRC), Adrienne New (CRC), Liliana Calderon (past CRC), Elmira Ramos (past CRC), Ramon Guardiola (past CRC), Bhavin Shah (past CRC), Lenis Sosa (past CRC, Melissa Whipple (past CRC);
Medical University of South Carolina (4) - Alejandro Spiotta (Current PI and past Sub-I), Jonathan Lena (Sub-I), Ayesha Vohra (CRC), Meredith Robinson (CRC), Aquilla Turk (past PI), Mohamad Chaudry (past Sub-I), Kyle Fargen (past Sub-I), Raymond Turner (past Sub-I), Emily Young (past CRC), Adrian Parker (past CRC), Angela Robinson (past CRC), Andrew Dippre (past CRC), Anita Deveaux (past CRC), Amora Mayo-Perez (past CRC);
Baylor College of Medicine (3) - Peter Kan (PI), Bridget Solis (CRC), Melyssa Fink (CRC), Edward Duckworth (past Sub-I), Samantha Macias (CRC), Gilberto DeFreitas (CRC), Stephen Harold (CRC), Sree Vidya (past CRC);
Los Robles Regional (3) - Muhammad Asif Taqi (PI), Anastasia Vechera (CRC), Samuel Hou (past Sub-I), Sajid Suriya (past CRC), Syed Quadri (past CRC);
Methodist Healthcare Memphis (2) - Adam S. Arthur (PI), Lucas Elijovich (Sub-I), Daniel Hoit (Sub-I), Christopher Nickele (Sub-I), Amanda Nolte (CRC), Jessica Jameson (CRC), Barrett Patel (CRC), Hani Rashed (CRC), Jay Vachhani (past Sub-I), Vinodh Thomas Doss (past Sub-I)
Rush University (2) - Richard W. Crowley (Current PI and past Sub-I), Bartosz Jacher (CRC), Demetrius Lopes (past PI), Carol Macpherson (past CRC), Amanda Arand (past CRC), Christy Anton (past CRC), Michael Chen (past Sub-I), John Dao (past CRC), Francisco Acosta (past CRC);
Harborview Medical Center (2) - Danial Hallam (PI), Basavaraj Ghodke (Sub-I), Michael Levitt (Sub-I), Kellie Sheehan (CRC), Louis Kim (Sub-I);
SSM DePaul Health Center (2) - Richard Callison (PI), Amer Alshekhlee (Sub-I), Michelle Raymond (CRC), Sushant Kale (past Sub-I);
Vanderbilt University (1) - Michael Froehler (PI), Matt Fusco (Sub-I), Rohan Chitale (Sub-I), Drew Anderson (CRC), Natalie Hall (CRC), Sally (Sarah) Baggette (CRC), Dima Sbenaty (past CRC), Kathryn McNabb (past CRC), Morgan A. Pittman (past CRC), Joy Grabenstein (past CRC), David McKeel (past CRC);
